# A Hamster Model of Diet-Induced Obesity for Preclinical Evaluation of Anti-Obesity, Anti-Diabetic and Lipid Modulating Agents

**DOI:** 10.1371/journal.pone.0135634

**Published:** 2015-08-12

**Authors:** Louise S. Dalbøge, Philip J. Pedersen, Gitte Hansen, Katrine Fabricius, Henrik B. Hansen, Jacob Jelsing, Niels Vrang

**Affiliations:** Gubra, Hørsholm, Denmark; Monash University, AUSTRALIA

## Abstract

**Aim:**

Unlike rats and mice, hamsters develop hypercholesterolemia, and hypertriglyceridemia when fed a cholesterol-rich diet. Because hyperlipidemia is a hallmark of human obesity, we aimed to develop and characterize a novel diet-induced obesity (DIO) and hypercholesterolemia Golden Syrian hamster model.

**Methods and Results:**

Hamsters fed a highly palatable fat- and sugar-rich diet (HPFS) for 12 weeks showed significant body weight gain, body fat accumulation and impaired glucose tolerance. Cholesterol supplementation to the diet evoked additional hypercholesterolemia. Chronic treatment with the GLP-1 analogue, liraglutide (0.2 mg/kg, SC, BID, 27 days), normalized body weight and glucose tolerance, and lowered blood lipids in the DIO-hamster. The dipeptidyl peptidase-4 (DPP-4) inhibitor, linagliptin (3.0 mg/kg, PO, QD) also improved glucose tolerance. Treatment with peptide YY_3-36_ (PYY_3-36_, 1.0 mg/kg/day) or neuromedin U (NMU, 1.5 mg/kg/day), continuously infused via a subcutaneous osmotic minipump for 14 days, reduced body weight and energy intake and changed food preference from HPFS diet towards chow. Co-treatment with liraglutide and PYY_3-36_ evoked a pronounced synergistic decrease in body weight and food intake with no lower plateau established. Treatment with the cholesterol uptake inhibitor ezetimibe (10 mg/kg, PO, QD) for 14 days lowered plasma total cholesterol with a more marked reduction of LDL levels, as compared to HDL, indicating additional sensitivity to cholesterol modulating drugs in the hyperlipidemic DIO-hamster. In conclusion, the features of combined obesity, impaired glucose tolerance and hypercholesterolemia in the DIO-hamster make this animal model useful for preclinical evaluation of novel anti-obesity, anti-diabetic and lipid modulating agents.

## Introduction

Obesity has become an increasing health concern worldwide due to the alarming rise in prevalence [1]. Along with obesity development there is an increase in obesity related complications, increased mortality and increase in health care expenses [2]. The major obesity related co-morbidities are impaired glucose tolerance, decreased insulin sensitivity, and dyslipidemia—all being risk factors for cardiovascular diseases [2].

The availability of animal models reflecting the human setting is essential for the development of pharmacological treatments. Several genetic animal models mimicking an obese/diabetic phenotype are already available; including the homozygous db/db mouse and Zucker diabetic fatty rats (ZDF) which carries a genetic defect in the leptin receptor [3, 4]. Although these animals are important for obesity and type 2 diabetes research, genetically modified animal models also provide a number of limitations, and they do not mimic the complicated interaction of polygenetic and various environmental factors involved in the development of obesity and obesity related complications in humans[4, 5].

We have recently phenotyped a novel rat model of diet-induced obesity (DIO), which shows marked obesity and development of impaired glucose tolerance when offered a two-choice diet of regular chow pellets and a highly palatable fat- and sugar-rich diet (HPFS) [6]. However, even though the DIO rat is a popular model for the metabolic syndrome the DIO rat only reflects the obese and insulin resistant phenotypes. The plasma lipoprotein profile in the rat (and mouse) is mainly based on High-density lipoproteins (HDL) and does not resemble the human phenotype where circulating lipoproteins primarily contains low-density lipoproteins (LDL) [7, 8]. Importantly, rats and mice are resistant to develop hypercholesterolemia even on high cholesterol diets [8]. Collectively, these rodent DIO models are not applicable for studying drug effects on hyperlipidemia. Alternative genetically modified animal models are available such as the apolipoprotein E (apoE)-deficient mice. Transgenic ApoE mice develop severe hyperlipidemia with elevated very low-density lipoproteins (VLDL) and decreased HDL levels but do not become obese or glucose intolerant when fed a high-fat diet [7]. Hence, while several animal models are dealing with either obesity related impaired glucose tolerance or dyslipidemia none of these models resemble the human setting where obesity and impaired glucose tolerance often goes along with hyperlipidemia. The development of such a pre-clinical model could be of substantial importance in the search for new effective therapies against the human metabolic syndrome.

The Golden Syrian hamster has previously been used widely in studies of lipoprotein metabolism [9, 10]. Unlike rats and mice, Golden Syrian hamsters have an atherogenic lipoprotein profile with a large proportion of the circulating lipoproteins being the non-HDL form, they possess cholesteryl ester transport protein (CETP), receptor mediated uptake of LDL lipoproteins via the LDL receptor, exclusively hepatic production of apolipoprotein (apo) B-100 and intestinal production of apo B-48 [8–13]. Accordingly, hamsters quickly develop hypercholesterolemia and hypertriglyceridemia when fed a cholesterol-rich diet [9, 14, 15]. In addition, it has previously been demonstrated that hamsters are obesity prone and develop insulin resistance when fed a high fat-high carbohydrate diet [16–18]. Collectively, these features indicate that the hamster may be an excellent species for the assessment of drugs with combined efficacy on weight loss, glucose tolerance, hypertriglyceridemia and hypercholesterolemia.

We therefore developed a DIO-hamster model with combined characteristics of impaired glucose metabolism, hypertriglyceridemia and hypercholesterolemia. Importantly, this model showed a marked responsiveness to anti-obesity, anti-diabetic and lipid-lowering drugs commonly used in the clinic. Including liraglutide (glucagon-like peptide-1 (GLP-1) receptor agonist [19]), linagliptin (dipeptidyl peptidase-4 (DPP-4) inhibitor [20]), and ezetimibe (cholesterol uptake inhibitor [21]). Furthermore, we characterized the effect of the two peptides peptide YY_3-36_ (PYY_3-36_) and neuromedin U (NMU), which both have recently been reported to induce robust anorectic and body weight lowering properties in other rodent DIO models [22, 23].

## Materials and Methods

### Animals

All animal experiments were conducted in strict accordance with internationally accepted principles for the care and use of laboratory animals and were covered by a personal license issued for Jacob Jelsing (approved by the Danish Committee for Animal Research, permit number: 2013-15-2934-00784).

Male Golden Syrian hamsters, (*mesecricetus auratus*, HsdHan:AURA) six weeks of age were obtained from Harlan Laboratories (Indianapolis, IN, USA) and housed five per cage under a 12:12 hour light-dark cycle, lights on at 04:00 AM. The room temperature was controlled to 20–22 degrees Celcius and the relative humidity to 50–60%. To test the susceptibility of Golden Syrian hamster to diet induced obesity and hyperlipidemia a variety of different diets containing variable amounts of fat, sugar and free cholesterol were tested (Table 1). Eventually, hamsters were offered either a regular rodent chow (#1324, Altromin, Lage, Germany), a high-fat diet (D12266B 31.8E%fat or D12492 60.0E%fat, Research Diets, New Brunswick, NJ, USA), or a two-choice diet consisting of regular rodent chow and a highly palatable fat- and sugar-rich (HPFS) diet made from equal amounts of chocolate spread (Nutella, Ferrero, Pino Torinese, Italy), peanut butter (Skippy Creamy, Hormel Foods, Austin, MN, USA) and powdered rodent chow (40.3E%fat) with or without 0.5% cholesterol to test the effect of cholesterol on circulating blood lipids. All animals had free access to tap water (Table 1).

**Table 1:** Energy content of diets used to induce obesity and hyperlipidemia.

Diet	D12266B	D12492	HPFS	HPFS + chol.
pr. 100g				
% Energy from fat	31.8E% Fat	60E% Fat	40.3E% Fat	40.3E% Fat
Energy (kcal)	441	524	485	485
Energy (kJ)	1845	2192	2032	2032
Fat (g)	15.6	34.9	29.3	29.3
Carbohydrate (g)	56.7	26.3	33.2	33.2
Protein (g)	18.5	26.2	18.0	18.0
Cholesterol (g)	-	-	-	0. 5

Body weight (BW) was measured weekly in feeding studies and daily in treatment studies. Food intake was measured daily throughout the treatment studies. All animals were euthanized after the study by CO_2_/O_2_ anesthesia and trunk blood was collected and analyzed for total plasma triglycerides (TG), total cholesterol, high density lipoproteins (HDL) and low density lipoproteins (LDL). During the feeding period all blood samples were sampled from free-fed animals. In the treatment studies animals were semi-fasted before blood sampling as they had access to only 50% of their average 24-hour intake (given at 16:00PM the day before the test). Cages were changed at the time of fasting and chin pouches were checked for food.

### Drug treatment

Hamsters included in treatment studies were fed a HPFS diet with 0.5% cholesterol supplemented for 12 weeks. Hereafter, the hamsters were stratified based on body weight before start of intervention. Vehicle for subcutaneous (SC) dosing consisted of phosphate buffered saline (PBS) added 0.1% bovine serum albumin (BSA). Vehicle for oral dosing consisted of 0.5% hydroxypropyl methylcellulose (HPMC). Vehicle for osmotic pumps consisted of 0.9% saline. Liraglutide (Victoza, Novo Nordisk, Bagsvaerd, Denmark) was dosed 0.2 or 0.1 mg/kg SC twice a day (BID) for 27 or 14 days, administered 2 and 10 hours into the light phase. Linagliptin (Trajenta, Boehringer Ingelheim, Ingelheim am Rhein, Germany) was dosed 3.0 mg/kg *per os* (PO) once a day (QD) for 27 days two hours into the light phase. Peptide YY_3-36_ (PYY_3-36_) and neuromedin U (NMU) were delivered continuously (2.5μl/h, 1.0 and 1.5 mg/kg/day, respectively) by an implantable subcutaneous osmotic minipump (model 2ML4, Alzet, Cupertino, CA, USA) for 14 days. Ezetimibe (Ezetrol, MSD Denmark, Ballerup, Denmark) was dosed 10.0 mg/kg PO QD for 14 days two hours into the light phase. PO dosing was performed via oral gavage with a 20 gauge feeding tube (Instech, Plymouth Meeting, PA, USA). The first dose was given on day 0.

### Non-invasive whole body composition analysis

Whole-body fat mass was analyzed by non-invasive EchoMRI scanning (EchoMRI-900, Houston, TX, USA). During the scanning procedure, the hamster was placed in a restrainer for approximately one minute.

### Oral glucose tolerance test

An oral glucose tolerance test (OGTT) was performed after 26 days on animals treated with liraglutide and linagliptin. Animals were semi fasted (access to only 50% of their average daily energy intake in the 16 hours preceding the OGTT). An oral glucose load (2 g/kg, glucose: 500 mg/L, Fresenius Kabi, Uppsala, Sweden) was administered 45 minutes after dosing. Blood samples for blood glucose and plasma insulin analyses were collected from the sublingual capillaries (capillaries perforated using 23-gauge needle) at *t* = -60, 0, 15, 30, 60, and 120 minutes prior to and after the oral glucose load. Glucose and insulin area under the curve (AUC) calculations were determined as total AUC from the sampling period of 0 to 120 min.

### Blood chemistry analyses

#### Whole blood glucose

10 μL blood was collected in a heparinized glass capillary and immediately suspended in 0.5 ml lysis buffer (glucose/lactate system solution, EKF-diagnostics, Cardiff, Great Britain), and then analyzed for glucose using a BIOSEN c-Line glucose meter (EKF-diagnostics).

#### Insulin

A total of 100 μL of blood was collected in heparinized tubes. Plasma was separated and insulin was measured in duplicate for each data point using an ultrasensitive ELISA kit (Mercodia AB, Uppsala, Sweden).

#### Blood lipid analysis

A total of 200 μL of blood was collected in heparinized tubes and the plasma was separated. TG, total cholesterol, HDL, and LDL were measured using the autoanalyzer Cobas C-111 and a commercial kit developed for direct measurement of lipoproteins in human plasma (5401488190, 5401682190 and 4657594190, Roche Diagnostics, Mannheim, Germany). It should be noted that although direct measurement of plasma lipoproteins has been validated for human use by the National Institute of Health the direct method used to measure plasma lipoproteins is regarded less robust than separation of plasma lipoproteins by fast protein liquid chromatography [24].

### Statistical evaluation

To test the normal distribution of data residuals the Kolmogorov-Smirnov test was performed. Comparisons of BW, glucose and insulin response during an OGTT and food intake were made using a 2-way analysis of variance (ANOVA). Comparisons of blood lipid levels and AUC glucose and insulin of the OGTT were made using a 1-way ANOVA. If a significant difference was observed, a Bonferroni post hoc test was performed to compare treatment groups to controls. All data were analyzed using GraphPad Prism 5.0 and InStat software. The results are presented as mean ± SEM. A *P*-value lower than 0.05 was considered statistical significant.

## Results

### Diet-induced obesity and hypercholesterolemia in the Golden Syrian hamster

**Fig 1:**
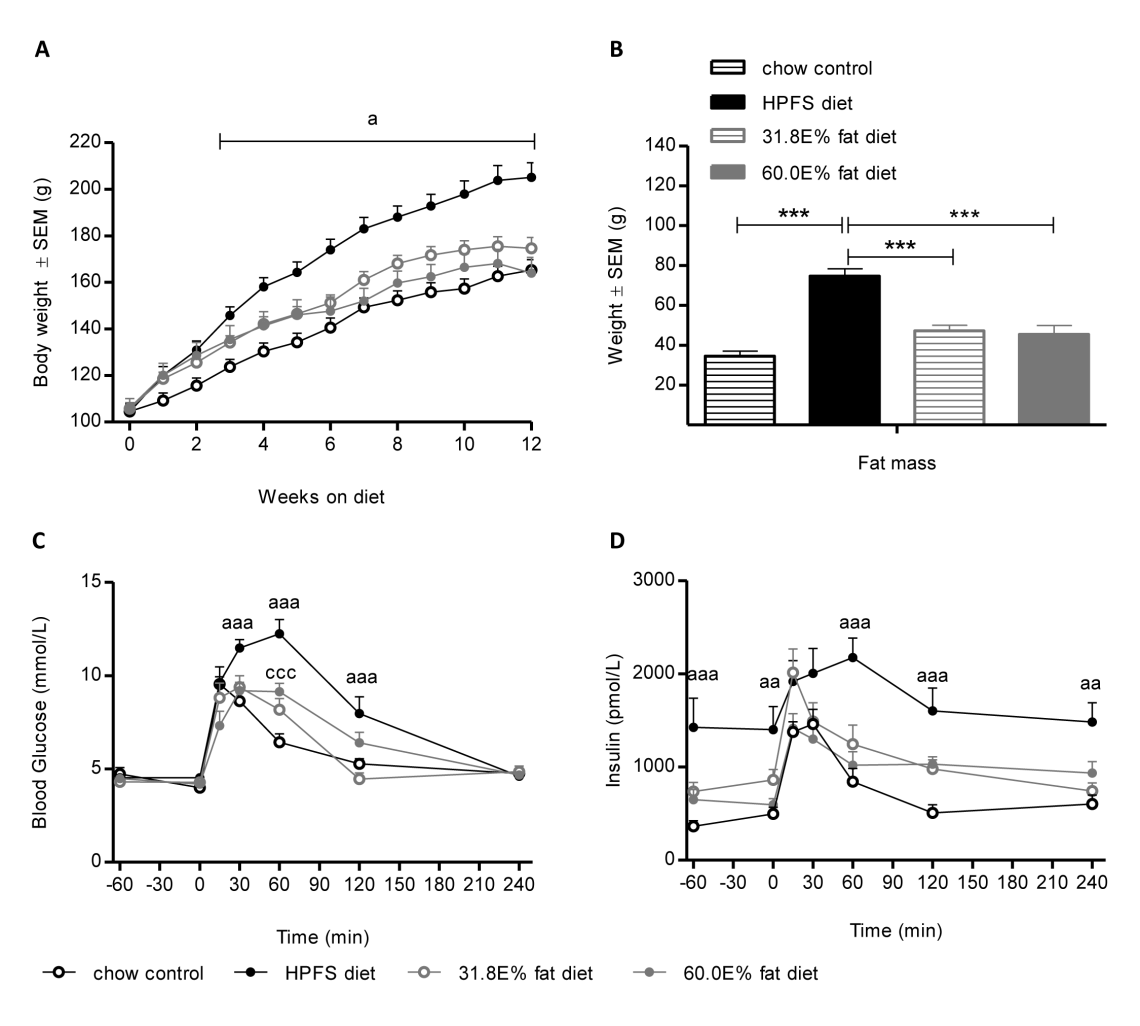
Hamsters fed a palatable diet become more obese than hamsters fed commercial purified high-fat diets. (A) Body weight (g), (B) whole-body fat mass (g), and (C) blood glucose and (D) plasma insulin levels were measured during an OGTT of hamsters fed chow, highly palatable fat- and sugar-rich (HPFS) diet, 31.8E % and 60.0E % purified HF diets for 12 weeks. Values are means ± SEM (n = 10 per group; ^a^P<0.05 for HPFS vs. chow-fed group and ^c^P<0.05 for 60.0E % HF diet vs. chow fed-group, (*** indicates *P*<0.001).

**Fig 2:**
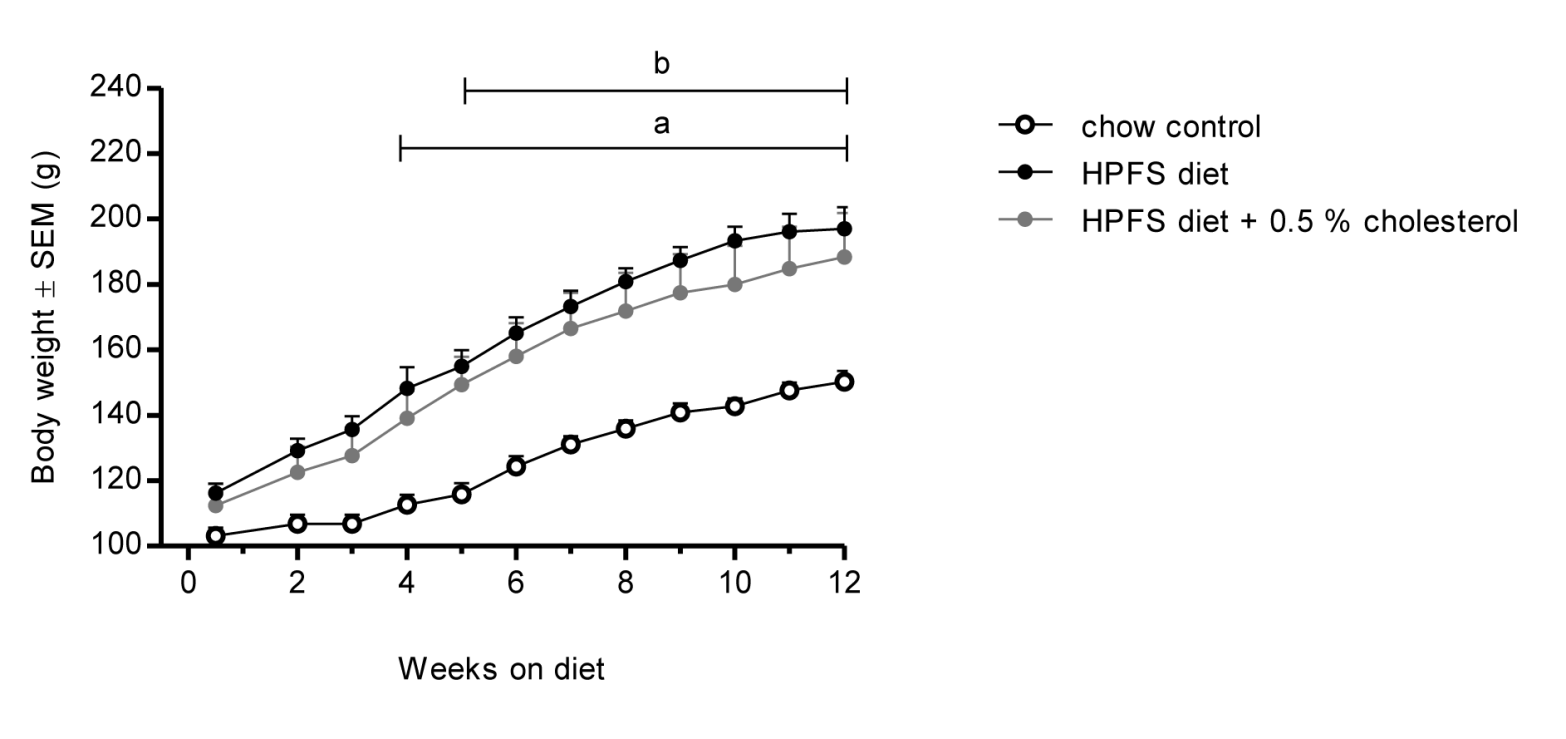
Body weight of hamsters fed highly palatable fat- and sugar-rich diet with 0.5% cholesterol supplementation. Body weight (g) of hamsters fed the highly palatable fat- and sugar-rich (HPFS) diet with 0.5% cholesterol supplementation for 12 weeks. Values are means ± SEM (n = 6 per group; ^a^P<0.05 for HPFS vs. chow-fed, ^b^P<0.05 for HPFS + 0.5% cholesterol vs. chow-fed group).

To test the susceptibility to diet induced obesity in Golden Syrian hamster a variety of different diets containing variable amounts of fat, sugar and free cholesterol were tested. Hamsters developed obesity when fed a highly palatable fat- and sugar-rich (HPFS) diet for 12 weeks, as compared to hamsters fed a regular rodent chow (p < 0.001) (Fig 1A). In addition, an increase in whole-body fat mass was observed both as compared to chow (p < 0.001) but also as compared to animals feed the purified high-fat diets (31.8E% fat, p < 0.001 and 60.0E% fat, p < 0.001) (Fig 1B). In contrast to the HPFS diet the purified high-fat diets (31.8E% fat and 60.0 E% from fat), only promoted a rather modest increase in body weight and whole-body fat accumulation. Additionally, HPFS diet induced hyperinsulinemia and impaired glucose tolerance in the DIO-hamster, which was not apparent in hamsters fed regular chow (Fig 1C and 1D). Neither the purified high-fat diets nor the HPFS diet significantly altered plasma lipids as compared to the chow fed group (data not shown). Accordingly 0.5% cholesterol supplementation was added to the diet in order to induce a more atherogenic plasma lipoprotein profile in the hamsters. Cholesterol supplementation to the HPFS diet did not significantly affect BW gain in DIO-hamsters (Fig 2), but lead to significantly increased levels of cholesterol and triglycerides in the DIO-hamster (p < 0.001, Table 2).

**Table 2:** Diet-induced obesity and hypercholesterolemia in the Golden Syrian hamster.

	Chow	HPFS	HPFS +0.5% col.
Whole-body fat mass (g)	27.13 ± 2.75	79.22 ± 4.58***	63.00 ± 13.85***
Triglycerides (mmol/l)	2.30 ± 0.18	2.07 ± 0.26	5.38 ± 0.27***
Total cholesterol (mmol/l)	2.74 ± 0.16	3.18 ± 0.28	7.74 ± 0.40***
HDL cholesterol (mmol/l)	1.77 ± 0.09	2.14 ± 0.08	4.71 ± 0.24***
LDL cholesterol (mmol/l)	0.59 ± 0.12	0.59 ± 0.13	1.58 ± 0.24***
LDL/HDL	0.33 ± 0.04	0.27 ± 0.04	0.33 ± 0.02
Insulin (pmol/L)	373.7 ± 82.3	1389 ± 147***	957.6 ± 157*

Whole-body fat mass (g), free-fed blood lipid and insulin levels of hamsters fed high fat-high carbohydrate (HPFS) palatable diet with 0.5% cholesterol supplementation for 12 weeks. Values are means ± SEM (n = 6 per group; *P*<0.05 (*), *P*<0.001 (***) vs. chow-fed hamsters).

### Effects of test compounds on body weight, insulin sensitivity, and blood lipids

**Fig 3:**
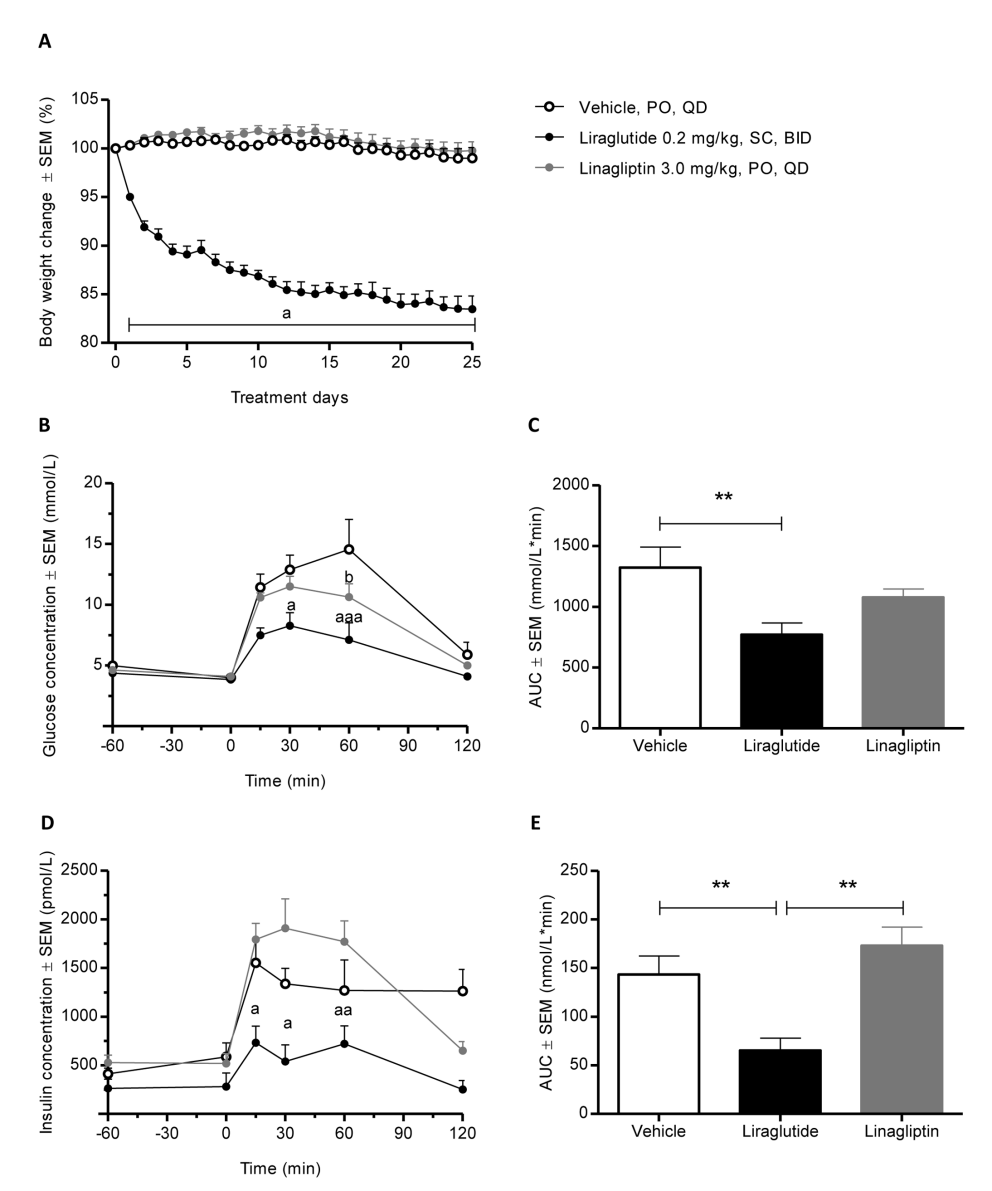
Effect of treatment with liraglutide and linagliptin on body weight and glycaemic control in DIO hamsters. (A) Body weight change (% of day 0) of obese hyperlipidemic hamsters treated for 26 days with vehicle (PO, QD), liraglutide (0.2 mg/kg, SC, BID) or linagliptin (3.0 mg/kg, PO, QD). Mean weight on day 0 was 185.6 ± 18.29 g. Absolute weight loss for the liraglutide group was 31.8 ± 14.6 g. (B, C) blood glucose and (D, E) plasma insulin responses to an OGTT following 26 days of treatment with vehicle (PO, QD), liraglutide (0.2 mg/kg, SC, BID) or linagliptin (3.0 mg/kg, PO, QD). Hamsters were semi-fasted 16 h before the OGTT. Drugs were administered 45 min prior to the oral glucose load (2g/kg). Hamsters included in the study were fed a HPFS diet with 0.5% cholesterol supplemented for 12 weeks before initiation of the study. Values are means ± SEM (n = 6 per group; ^a^P<0.05 for liraglutide vs. vehicle group, ^b^P<0.05 for linagliptin vs. vehicle group, (** indicates *P*<0.01).

Chronic treatment with the GLP-1 analogue, liraglutide (0.2 mg/kg SC, BID) reduced BW 16.5 ± 1.35% after 26 days of treatment (Fig 3A). Liraglutide significantly reduced blood glucose and plasma insulin levels over time during an OGTT (Fig 3B and 3D) leading to a near normalized postprandial glycaemic control. This was also evident when expressed as area under the curve (AUC) (Fig 3C and 3D). Also, liraglutide significantly lowered total triglyceride (p < 0.001), total cholesterol (p < 0.01), and HDL levels (p < 0.05) in the DIO-hamster (Table 3).

**Table 3:** Effect of liraglutide and linagliptin on body weight, and blood lipids.

	Vehicle	Liraglutide	Linagliptin
Whole-body fat mass (g)	50.10 ± 5.57	31.42 ± 4.80*	51.83 ± 3.79
Triglycerides (mmol/l)	3.78 ± 0.22	1.18 ± 0.12***	3.65 ± 0.40
Total cholesterol (mmol/l)	6.54 ± 0.40	4.14 ± 0.51**	5.46 ± 0.29
HDL cholesterol (mmol/l)	5.22 ± 0.33	3.58 ± 0.46*	4.17 ± 0.34
LDL cholesterol (mmol/l)	0.76 ± 0.13	0.50 ± 0.14	0.52 ± 0.07
LDL/HDL	0.14 ± 0.02	0.12 ± 0.02	0.12 ± 0.09

Whole-body fat mass (g) and semi-fasted blood lipid levels of obese hyperlipidemic hamsters treated for 27 days with vehicle (PO, QD), liraglutide (0.2 mg/kg, SC, BID) or linagliptin (3.0 mg/kg, PO, QD). Values are means ± SEM (n = 6 per group; P<0.05 (*); P<0.01 (**); P<0.001 (***) vs. vehicle group).

Linagliptin also improved glucose tolerance in the DIO-hamster, however, less pronounced as compared to liraglutide. In contrast to liraglutide, linagliptin administration had no effect on body weight gain, blood lipid levels, as well as OGTT insulin levels in the DIO-hamster (Fig 3 and Table 3).

**Fig 4:**
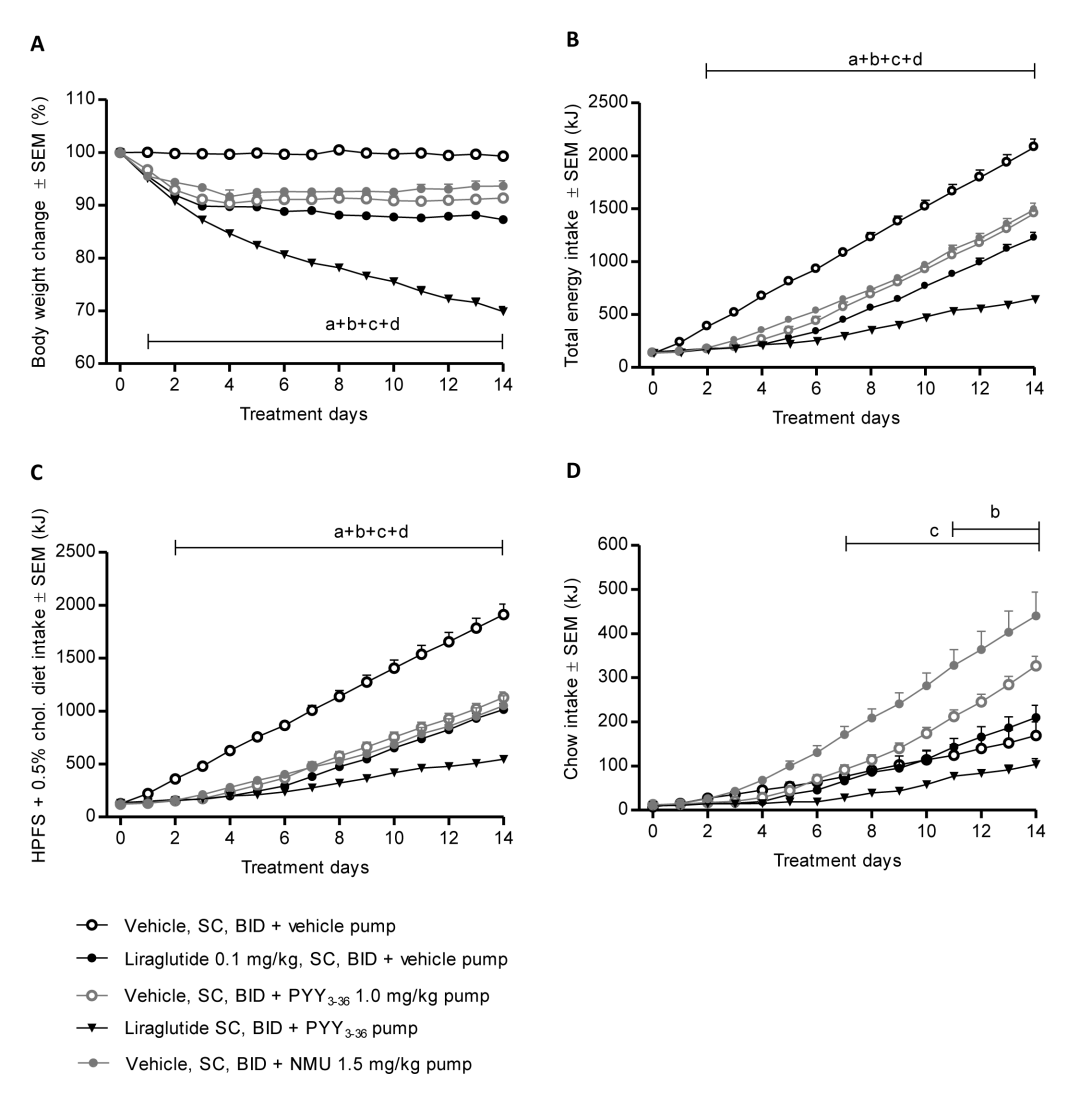
Effect of NMU, PYY_3-36_, liraglutide or co-treatment with liraglutide and PYY_3-36_ on body weight and cumulative energy intake. (A) Body weight change (% of day 0), (B) Cumulative total energy intake (kJ), (C) cumulative intake of the HPFS diet with 0.5% cholesterol supplement (kJ), and (D) cumulative intake of regular chow (kJ) after 14 days treatment of obese hyperlipidemic hamsters with vehicle (SC, BID) + vehicle (osmotic pump), liraglutide (0.1 mg/kg, SC, BID) + vehicle (osmotic pump), vehicle (SC, BID) + PYY_3-36_ (1.0 mg/kg/day, osmotic pump), the co-treatment group liraglutide (0.1 mg/kg, SC, BID) + PYY_3-36_ (1.0 mg/kg/day, osmotic pump), or vehicle (SC, BID) + NMU (1.5 mg/kg/day, osmotic pump). Hamsters included in the study were fed a HPFS diet with 0.5% cholesterol supplemented for 12 weeks before initiation of the study. Data are given as mean ± SEM with n = 8/group. Statistical analysis: two-way ANOVA with Bonferronis post hoc test; ^a^ *P*<0.05 for liraglutide vs. vehicle group, ^b^ *P*<0.05 for PYY_3-36_ vs. vehicle group, ^c^ *P*<0.05 for PYY_3-36_ + liraglutide vs. vehicle group, and ^d^ *P*<0.05 for NMU vs. vehicle group.

Treatment with PYY_3-36_ and NMU for 14 days reduced body weight by 8.3 ± 0.6% and 6.7 ± 1.1%, respectively, in the DIO-hamster. In comparison, 14 days of liraglutide treatment reduced body weight by 12.5 ± 0.4% (Fig 4A). Combined PYY_3-36_ and liraglutide treatment evoked a pronounced and supra-additive reduction in body weight with no apparent lower plateau being established after 14 days of treatment, at which time a body weight loss of 32.3 ± 1.74% was observed. The reductions in body weight reflected an immediate suppression of total energy intake (Fig 4B). Interestingly, NMU and PYY_3-36_ induced a shift in food preference with a significant decrease in the palatable HPFS diet accompanied by a significant increase in chow consumption (Fig 4C and 4D).

Finally, treatment with the cholesterol uptake inhibitor ezetimibe for 14 days significantly lowered plasma total cholesterol (p < 0.001), HDL (p < 0.01), and LDL (p < 0.001) levels in the DIO-hamster (Table 4). The effect of ezetimibe on plasma LDL levels was more marked as compared to HDL levels which was reflected in a significant reduction in the LDL/HDL ratio.

**Table 4:** Effect of ezetimibe on blood lipids.

	Vehicle	Ezetimibe
Triglycerides (mmol/l)	2.04 ± 0.17	1.94 ± 0.14
Total cholesterol (mmol/l)	6.62 ± 0.16	5.37 ± 0.22***
HDL cholesterol (mmol/l)	5.81 ± 0.15	4.76 ± 0.20**
LDL cholesterol (mmol/l)	1.26 ± 0.09	0.75 ± 0.08***
LDL/HDL	0.22 ± 0.01	0.16 ± 0.01**

Semi-fasted blood lipid levels of obese hyperlipidemic hamsters treated for 14 days with vehicle (PO, QD) or Ezetimibe (10.0 mg/kg, PO, QD). Values are means ± SEM (n = 8 per group; P<0.01 (**); P<0.001 (***) vs. vehicle group).

## Discussion

In the present study we validate the DIO-hamster as a useful model of combined obesity, glucose intolerance hypertriglyceridemia and hypercholesterolemia, all being important features of the human obesity syndrome. Importantly, we demonstrate a high sensitivity to a number of drugs having well-established anti-obesity, anti-diabetic and lipid lowering efficacy in humans.

In particular, rodent DIO models where animals have a choice between regular chow and a highly palatable diet, typically consisting of chocolate and other energy dense foods more similar to western (‘cafeteria’) diets, have demonstrated their relevance as valid models of human obesity [25]. Such diet compositions promote voluntary hyperphagia and induce obesity to a higher degree than standard purified high fat diets [25–27]. In agreement with this view, we show that hamsters fed a palatable diet consisting of chocolate spread, peanut butter and chow were markedly more obese than hamsters fed commercial purified high-fat diets. Previous reports have indicated that Golden Syrian hamsters are highly susceptible to dietary cholesterol which promotes robust hypercholesterolemia [13, 15]. As a result, we provided 0.5% cholesterol supplementation to the diet in order to induce a more atherogenic plasma lipoprotein profile in the hamsters. The procedure evoked an additional hypercholesterolemia and hypertriglyceridemia in the DIO-hamster without affecting the marked obesity status of the DIO-hamsters. Similar to mouse and rat DIO models, the DIO-hamster did not develop overt diabetes but did display impaired glucose tolerance and hyperinsulinemia. Treatment with liraglutide and linagliptin improved glucose tolerance in an oral glucose tolerance test, but only liraglutide reduced body weight, being in close agreement with previously published reports in other DIO models [6, 28, 29].

We further validated the model by testing the effects of the two peptides PYY_3-36_ and NMU. PYY_3-36_ is a gut-derived hormone which is secreted postprandial by endocrine L-cells and is known to decrease food intake and body weight in mouse and rat DIO models [23, 30]. The neuropeptide NMU is expressed and secreted in both the brain and the gut, and has been suggested also to be implicated in energy and appetite regulation [22]. In the present study, we confirmed that both PYY_3-36_ and NMU also have body weight lowering properties in DIO-hamsters.

Because combination therapies are emerging as important new treatment strategies against the metabolic syndrome [31–33], we investigated the effect of combined liraglutide and PYY_3-36_ administration in the DIO-hamster. Interestingly, subcutaneous infusion of PYY_3-36_ in combination with subcutaneous liraglutide treatment caused a marked synergistic effect on body weight and food intake reduction. In contrast to liraglutide and PYY_3-36_ monotherapy, a continuous weight loss was observed in the drug combination group and no lower plateau was reached by the end of the treatment period. These findings are in agreement with previous reports showing that co-treatment with native GLP-1_7−36_ and PYY_3-36_ additively suppress food intake in rodent and humans [34]. Interestingly NMU, PYY_3-36_ and liraglutide treatment also changed food preference in the DIO-hamster, as demonstrated by the marked increase in chow intake at the expense of the palatable HPFS diet consumption. Alterations in food preference have previously been reported for liraglutide in DIO rats [6, 28, 29], and both PYY and GLP-1 have also been suggested to be involved in hedonic regulation of food intake in humans [35, 36]. Furthermore, it has been reported that intracerebroventricular administration of NMU increases chow preference whereas knock down of the NMU receptor in the paraventricular nucleus (PVN) increases the preference for dietary fat [37, 38]. Accordingly, the present DIO-hamster model allows for the detection of changes in diet preferences whereas the standard obesity models, fed a regular high-fat diet, would fail to detect such a parameter.

The hamster has been widely used in studies of lipoprotein metabolism and diet-induced atherosclerosis as their lipoprotein metabolism closer resembles the human conditions than mice and rats [9, 10]. However, in the present study we failed to detect the development of aortic lesions (data not shown), indicating that this DIO-hamster model does not appear to be a useful model to study the potential pharmacological effect on development of atherosclerosis. In agreement, inconsistent results have been reported previously on diet-induced atherosclerosis and aortic lesions in the Golden Syrian hamster (reviewed in details by Dillard and colleagues [9]). Although, we cannot exclude the possibility that higher concentrations of supplementary cholesterol may have increased aortic accumulation of cholesterol and thus could potentially induce aortic lesions in our models as reported previously by others [10, 15]. However, it should be noted that additional supplementation of cholesterol to the diet is likely incompatible as dietary cholesterol levels of 1%, or higher, is hepatotoxic and decrease body weight in Golden Syrian hamsters [9, 15]. The lipoprotein profile observed in the golden hamster (with HDL concentrations slightly higher than LDL concentrations) is in agreement with other reports [9] but in contrast to the human metabolic phenotype where LDL cholesterol is elevated and HDL cholesterol is low [39]. Accordingly, the present hamster model does not completely reflect the human setting and the less atherogenic lipoprotein profile could potentially explain the lack of atherosclerosis development in the hamster model. In this respect, however, it should be noted that the lipoprotein system of hamsters have a higher resemblance to humans, than that of rats, due to a larger proportion of the circulating lipoproteins being the non-HDL form, the possession of CETP and receptor mediated uptake of LDL lipoproteins via the LDL receptor [8–13]. Finally, the effect of the cholesterol absorption inhibitor ezetimibe (a lipid lowering drug commonly used in the clinic) was tested to assess the responsiveness of plasma cholesterol in DIO-hamster. Treatment with ezetimibe reduced plasma total cholesterol without affecting body weight, and with a more marked reduction of LDL levels compared to HDL. These data are in accordance with previously reported results in hyperlipidemic DIO-hamsters and indicates sensitivity to cholesterol modulating drugs [40].

Novel treatment strategies are required to tackle the continuous growing obesity problem and the obesity related complications. Accordingly, reliably animal models reflecting the human metabolic syndrome are essential. In the present study we have validated a DIO-hamster model as a useful polygenic model closely reflecting core aspects of the human metabolic syndrome. The DIO-hamster rapidly develops an obesity-like phenotype accompanied by impaired glucose tolerance, hyperinsulinemia, hypertriglyceridemia, and hypercholesterolemia when fed a HPFS diet with 0.5% supplementary cholesterol whereas mice and rats only develop the obese and hyperinsulinemic phenotype. Furthermore, the model responds effectively to drugs with well-established anti-obesity, anti-diabetic and lipid-lowering efficacy. Accordingly, our study suggests the feasibility of using the DIO-hamster as compared to other animal models of obesity. Also, for a practical point of view, the hamster is easy to handle and substantial amounts of blood can be drawn from the DIO-hamster as compared to mouse models. Furthermore the DIO-hamster is smaller than the DIO rat (200g vs 800g) and accordingly less amount of compound is required during preclinical development.

In conclusion, the characteristics of combined obesity, impaired glucose tolerance and hypercholesterolemia in the DIO-hamster make this animal model useful for preclinical evaluation of novel anti-obesity, insulin sensitizing, and lipid modulating agents.

## Supporting Information

S1 File The supporting information file contains all the relevant data for the included figures (Figs 1, 2, 3 and 4) and tables (Tables 1–4). The data are presented in the excel spreadsheet.
(XLS)
